# Characterization of Acne-Prone Skin with Reflectance Confocal Microscopy and Optical Coherence Tomography and Modifications Induced by Topical Treatment and Probiotic Supplementation

**DOI:** 10.3390/jcm12144787

**Published:** 2023-07-20

**Authors:** Marco Manfredini, Alberto Sticchi, Nicola Lippolis, Gioia Pedroni, Matteo Giovani, Silvana Ciardo, Camilla Chello, Stefania Guida, Francesca Farnetani, Giovanni Pellacani

**Affiliations:** 1 Dermatology Unit, Department of Surgical, Medical, Dental & Morphological Sciences with Interest Transplant, Oncological & Regenerative Medicine, University of Modena and Reggio Emilia, 41125 Modena, Italy; 2 Centro Oncologico ad Alta Tecnologia Diagnostica, Azienda Unità Sanitaria Locale—IRCCS, 42123 Reggio Emilia, Italy; 3 Dermatology Clinic, Department of Clinical Internal, Anesthesiological and Cardiovascular Sciences, Sapienza University of Rome, 00185 Rome, Italy; 4 Dermatology Clinic, Vita-Salute San Raffaele University, 20132 Milan, Italy

**Keywords:** acne-prone skin, acne, confocal microscopy, optical coherence tomography, topical treatment, probiotic supplementation

## Abstract

The evaluation of acne-prone skin and absent-to-mild acne is difficult because this condition is not associated with a clinically definable situation. Previous studies showed that apparently healthy skin in patients with previous episodes of acne shows microcomedos and infundibular hyperkeratosis upon reflectance confocal microscopy (RCM) evaluation. Our aim was to characterize the subclinical and microscopic characteristics of acne-prone skin by means of RCM and dynamic optical coherence tomography (D-OCT) and evaluate microscopic changes induced by treatment. A group of 20 patients received a daily combined treatment over a period of 3 months, consisting of probiotic supplementation with three strains of 10^9^ colony-forming units of Lactobacillus (*Lactobacillus reuteri, Lactobacillus casei* subsp. *rhamnosus, Lactobacillus plantarum*) and a combined topical product of azelaic and hydroxypinacolone retinoate (HPR). Clinical evaluations and non-invasive imaging acquisitions using VISIA^®^ System, RCM, and D-OCT were performed at baseline, and after 4 and 12 weeks. The total number of clinically evident non-inflammatory lesions decreased during treatment from 11.5 to 7.3 (*p* < 0.05). There was also an evident reduction in microscopic acne features at RCM and D-OCT, such as the number of small bright follicles, large bright follicles and vascular threshold density at 300 μm and 500 μm depths. The types and extent of microscopic alterations in acne-prone skin patients may not be evident by clinical scores. Patients with low investigator global assessment (IGA) grades are a heterogeneous population, characterized by different microscopic skin features. Acne-prone skin is susceptible to treatment, and RCM and D-OCT imaging are sensitive tools to objectively monitor subclinical skin changes.

## 1. Introduction

Acne vulgaris is a common inflammatory skin disease of the pilosebaceous unit, with an estimated prevalence of 9.4% of the global population and a peak incidence among teenagers [1]. The exact prevalence in different studies varies partly depending on the method of classification, but several epidemiological studies demonstrated that it is most common in adolescent teens, with boys most frequently affected by more severe forms of the disease [2,3]. A positive familial history is significantly more common in patients with moderate to severe acne than in patients with mild disease [2,3]. Patients present a pleomorphic clinical presentation, with clinically evident inflammatory lesions (papules, pustules and nodules) or non-inflammatory lesions (closed and open comedones). The pathogenesis of acne vulgaris is not fully clarified, although it is widely accepted that the following four key factors play a central role in the development of the disease: follicular hyperkeratinization, increased sebum production, follicular colonization by Cutibacterium acnes (*C. acnes*), and release of inflammatory mediators into the skin [4]. Patients affected by mild to moderate acne are prone to frequent relapses after discontinuation of topical or systemic treatment, with recurrence rates up to 52%, depending on the treatment administered and acne type and severity [5]. Acne-prone skin is defined by the propensity to develop comedones or other acne lesions, clinically characterized by greasiness, in the absence of inflammatory lesions. Recently, specific alterations have been described in acne-prone skin, consisting of increased keratinization of the infundibulum and dysfunctional sebocyte differentiation mechanism, altogether defining the subclinical status of acne-prone skin [6,7,8,9].

Hyperkertinization of the infundibulum is considered one of the most important events in the pathogenesis of acne, leading to the obstruction of the follicular ostium and accumulation of sebum, keratin and bacteria. As a result, the infundibulum dilates, configuring the “elementary lesion” of acne-prone skin: the microcomedo, a subclinical precursor of acne lesions [8,9,10]. The inflammatory process associated with acne could exacerbate hyperkeratinization and degrade the microcirculation network during inflammation. This can trigger a fibrotic process, ultimately leading to scarring [11,12]. Many factors contribute to the formation of microcomedones: androgenic stimulation, a pro-inflammatory milieu, with inflammatory mediators released into the skin, and the presence of *C. acnes*, a Gram-positive, anaerobic bacteria that normally occupies the hair follicles and sebaceous glands [4,12,13]. Since these phenomena are unpredictable and can rapidly evolve into florid acne without any preceding signal, a maintenance regimen is frequently recommended in acne patients during the remission phase of the disease, in order to avoid, delay or at least mitigate potential recurrence.

In our pursuit of effective treatment monitoring methods, we have turned our attention to novel imaging technologies. Monitoring of microcomedones in acne-prone skin can serve as an objective measure for treatment effectiveness evaluations [8,10]. Precise microscopic features of acne elementary lesions have been outlined with non-invasive high-resolution imaging techniques, including accurate quantifications for disease severity staging and long-term therapeutical efficacy [14,15,16,17,18]. Reflectance confocal microscopy (RCM) is a non-invasive imaging technique with nearly histologic resolution. Previously applied in oncologic and inflammatory skin disease diagnoses [19,20,21], RCM allows the visualization of acne-associated infundibular alterations and microcomedones. Optical coherence tomography (OCT) is a tool that provides cross-sectional 2D and 3D skin images, or the microvascular blood flow, when used in dynamic mode (D-OCT). OCT has a lower resolution but a higher penetration than RCM [6,22,23,24]. RCM and D-OCT allow in vivo visualization of sub-clinical acne lesions. Together, they have been used to define in vivo acne pathophysiology and assess the treatment efficacy [25,26].

Recently, several studies have described significant gut dysbiosis in acne patients and other inflammatory skin disorders [27,28,29,30,31]. It is hypothesized that the gut microbiome may have important implications for acne pathophysiology and treatment and that oral probiotics can play an effective role in managing acne by directly preventing the growth of opportunistic bacteria or by controlling inflammation [27]. In addition, it was demonstrated that supplementation with the probiotic strain Lactobacillus rhamnosus SP1 (LSP1) in adults with acne normalized the skin expression of genes involved in insulin signaling and improved the appearance of the skin [32]. Maintenance therapy is necessary to maintain acne remission, achieved with an initial successful treatment regimen, and to minimize the risk of relapse [5]. Therefore, oral probiotics, mostly of the genus Lactobacillus, are often prescribed in combination with active treatments and/or maintenance regimens during the disease remission phase [31].

We aim to characterize subclinical and microscopic characteristics of acne-prone skin with RCM and D-OCT, and to evaluate microscopic changes induced by a maintenance regimen of daily supplementation of oral probiotics in conjunction with a topical, combination acne treatment (azelaic and hydroxypinacolone retinoate (HPR)).

## 2. Materials and Methods

This observational study included patients with a previous history of facial acne and undergoing a combination probiotic and topical therapy maintenance regime, who were approached for study enrolment at the Dermatologic Unit of the University of Modena and Reggio Emilia between June 2020 and September 2021. Personal history of acne was defined as the previous presence of mild to moderate acne, requiring either topical or systemic pharmacological treatment, according to European guidelines [33]. Selection criteria specified revised Leeds score 1–2, investigator global assessment (IGA) grade 0–1 [34], total number of non-inflammatory lesions <16, and a complete absence of inflammatory acne lesions. Exclusion criteria specified patients (i) who received other acne or probiotic therapies within 6 months of the enrollment date, including over-the-counter acne treatments and prescribed acne medications, and being (ii) pregnant or breastfeeding. This study was conducted according to the Declaration of Helsinki and was approved by the local ethics committee (EC #927/2019), and all patients provided written informed consent for study inclusion.

The included patients received a three-month daily combined treatment, consisting of probiotic cap supplementation with three strains of 10^9^ colony-forming units of Lactobacillus (*L. reuteri, L. casei* subsp. *rhamnosus, L. plantarum*) and a combination of azelaic and HPR topical product. Baseline clinical assessment included a revised Leeds score, IGA) grade, and the number of inflammatory and non-inflammatory lesions. Clinical evaluations and non-invasive imaging acquisitions were performed at baseline and after 4 and 12 weeks (T0, T1, T2,) by two medical doctors (MM, NL) with expertise in acne, RCM and D-OCT.

Clinical image acquisition was performed using a VISIA^®^ System (Canfield Imaging Systems). RCM (Vivascope 1500^®^, Vivascope GmbH, Munich, Germany) and D-OCT (Vivosight^®^ Michelson Diagnostics Ltd., Maidstone, UK) image acquisition and analysis were performed according to previous studies dedicated to acne [14,35]. A skin target area of 4.5 × 4.5 mm on the right cheek where clinically evident acne was observed was chosen at baseline. The area was mapped out on a transparent plastic film, modeled on the face of the patient. RCM and D-OCT imaging were performed on the same skin area during the study.

RCM mosaics were evaluated according to previously described acne-associated RCM features, including the number of normal infundibula, small and large bright follicles, and inflammatory lesions (papules and pustules). Inflammatory lesions were defined as roundish areas with ill-defined borders, containing bright particles, inflammatory small bright cells, bright compact organized amorphous material, and inflammatory infiltrate at the periphery of the lesion [7]. D-OCT images were processed through 3D image reconstruction software, and previously described acne-associated D-OCT features, including sebaceous gland hypertrophy and vascular threshold density, were analyzed [23].

Clinical scores, RCM and D-OCT features were calculated at each time point, reported as mean (M) ± standard deviation (SD). The normality of data was assessed through D’Agostino and Pearson normality testing. Student’s *t*-test (pairwise) was used to compare changes during the study for continuous variables. The Wilcoxon matched-pairs signed rank test was performed for revised Leeds, IGA, RCM inflammation, and OCT Sebaceous gland hypertrophy scoring, which are semi-quantitative variables (Prism 6.0, GraphPad Software, 225 Franklin Street, Boston, MA, USA). *p* < 0.05 was considered significant. Bonferroni correction was performed to evaluate multiple testing biases.

## 3. Results

Our study included 20 patients, of an average age of 21 years (18–24), most of whom were female (85%, *n* = 17) and Caucasian (90%, *n* = 18). Very mild acne (IGA 1), with few comedos (ranging from 9 to 15) and absent inflammatory facial lesions, was registered for 18 patients (90%). No inflammatory or non-inflammatory acne lesions at baseline (IGA 0) were registered for two patients who referred the recurrent, spontaneous appearance of some facial acne lesions. The baseline mean revised Leeds score was 1.45 (±0.51), with an IGA grade of 0.90 (±0.31); see Table 1.

**Figure 1:**
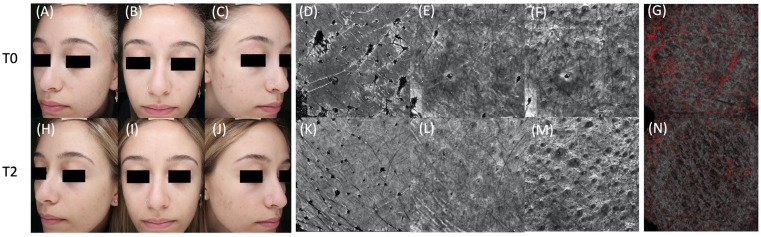
Clinical, reflectance confocal microscopy (RCM) and dynamic optical coherence tomography (D-OCT) images of a patient with acne-prone skin at baseline (**A**–**G**). Clinical images (**A**–**C**) show the presence of absent to minimal acne, with few visible comedos. Baseline RCM mosaic images obtained from a target area (4.5 mm × 4.5 mm) at the spinosus layer (**D**), at the dermal–epidermal junction (**E**) and at the upper dermis (**F**). Baseline RCM mosaic images (**D**–**F**) show the presence of many dilated infundibula and many small bright circles (microcomedos). Baseline D-OCT image (**G**) shows normal vascular signal. After 12 weeks of probiotic supplementation associated with an azelaic-based topical product, a significant reduction in non-inflamed comedos (**H**–**J**) and normalization of the RCM infundibular features is observed (**K**–**M**). A reduction of the D-OCT vascular signal can be observed (**N**).

**Figure 2:**
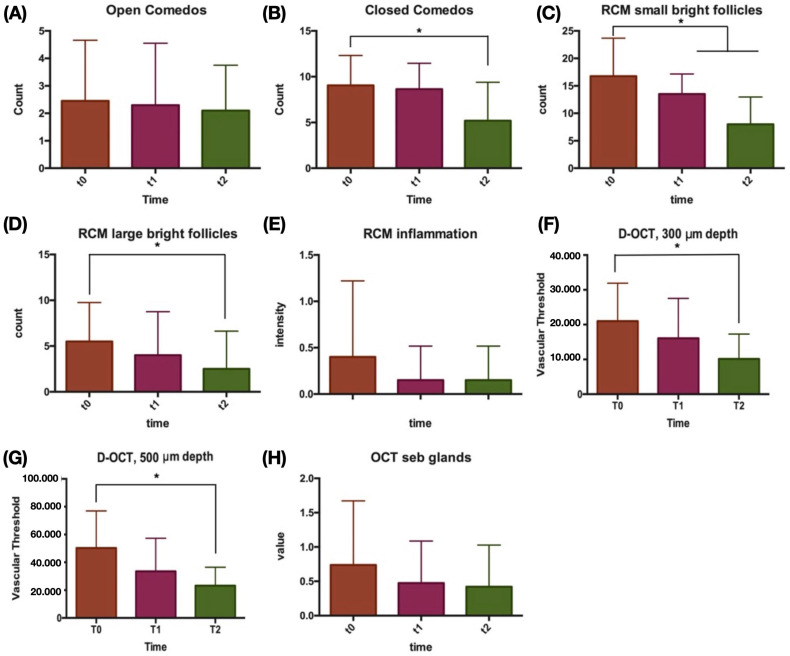
Vertical bar graphs showing the average values of clinical, reflectance confocal microscopy (RCM) and dynamic optical coherence tomography (D-OCT) parameters according to treatment time (T0, T1, T2). Close comedo number (**A**), open comedo number (**B**), RCM small bright circles (**C**), RCM large bright circles (**D**), RCM inflammation (**E**), D-OCT vascular signal intensity at 300 μm depth (**F**), D-OCT vascular signal intensity at 500 μm depth (**G**) and OCT enlarged sebaceous glands (**H**) are represented. Asterisks (*) denote *p* < 0.05 significative comparisons.

Clinically, the total number of non-inflammatory lesions significantly decreased during treatment, from 11.5 to 7.3, *p* < 0.05. With RCM evaluation, a significant reduction in small bright follicles was observed between baseline and both T1 and T2, *p* < 0.05. A significant reduction in large bright follicles was registered between T0 and T2, *p* < 0.05. By D-OCT evaluation, vascular thresholds at 300 μm and 500 μm were both significantly reduced by T2, *p* < 0.05. After Bonferroni correction, *p* < 0.002 was confirmed for the comparison between baseline and T2 of non-inflammatory lesions, closed comedos and small bright follicles. Differences in lesion counts, RCM and D-OCT parameters were normally distributed according to D’Agostino and Pearson testing. Additionally, a trend towards mean sebaceous gland hypertrophy reduction was observed throughout the follow-up; see Figure 1 and Figure 2.

## 4. Discussion

Our study highlights that acne-prone skin on a maintenance regime of daily supplementation with probiotic strains of *Lactobacillus* and topical application of a combination of azelaic and HPR for a 12-week period is characterized by subclinical alterations. These alterations are evident with RCM evaluations (small and large bright follicles and inflammatory features), and OCT (high vascular threshold density at 300 and 500 um, and sebaceous gland hypertrophy). During follow-up, the number of non-inflammatory lesions and all RCM and OCT features improved either significantly or by trend, whilst clinical scores were not significantly modified.

A universally accepted definition of maintenance therapy is still lacking. Several studies have investigated the efficacy of maintenance therapy in controlling acne relapse after successful initial treatments, even though the definition of recurrence is either lacking or different and the baseline severity of acne patients varies greatly [5,36]. Acne relapses have a negative psychological impact on patients, as they feel incapable of controlling the disease and achieving complete and persistent healing. In fact, acne may profoundly impair quality of life by limiting self-esteem and self-confidence, placing individuals at a significantly increased risk for developing psychiatric conditions such as depression and anxiety [5,37].

In this study, the acquisition of RCM and D-OCT images enabled a better qualitative and quantitative analysis of the microscopic alterations that characterize acne-prone skin and allowed the evaluation of the microscopic and functional changes in pilosebaceous-unit features induced by the maintenance regime. We observed great variability in microscopic aspects of acne-prone skin, with infundibular hyperkeratinization varying from 9 to 34 involved follicles. Similarly, RCM inflammatory patterns and D-OCT vascularization values showed wide standard deviations at baseline. Overall, these data suggest that the types and extent of microscopic alterations in acne-prone skin patients are likely to be underestimated by clinical scores.

It has already been demonstrated that comedogenesis is a complex process, usually characterized by a hyperkeratinizing stage followed by a vascular and inflammatory reaction [7,9]. Notably, among the 10 patients with more small bright follicles than the study average (*n* > 16), we observed that 4 had vascular thresholds below the study average values (mean < 21,030 (at 300 μm), <50,319 (at 500 μm)). This preliminary observation, if confirmed by larger studies, may have several implications. Firstly, we can hypothesize that patients with acne IGA grades 0–1 are a heterogeneous population, characterized by different microscopic skin features. Secondly, it may suggest that the progression from microcomedos to comedos and then to inflammatory lesions is not predetermined and may be strongly dependent on the balance between pro-tolerogenic and pro-inflammatory mechanisms. Further research is needed to explore these concepts that, in our opinion, are of the utmost importance in acne research.

The progressive reduction in the total count of comedones was correlated with the improvement of RCM and D-OCT parameters associated with acne during the maintenance regime. Small and large bright follicles correspond to infudibular hyperkeratinization and are the RCM features that have been associated with early presentation, leading to microcomedo development [4]. HPR is a retinoid with well-known properties, capable through its binding to RAR receptor of reducing infundibular hyperkeratinization and creating an aerobiotic microenvironment, hostile to *C. acnes*. Azelaic acid is a tyrosinase inhibitor and is widely used in acne treatment for its comedolytic and antibacterial properties.

As retinoids and azelaic acid are mild skin-irritating molecules [38], the daily supplementation of oral probiotics were prescribed to act synergically with the topical acne treatment to reduce inflammation. Active inflammation is not a typical feature of acne-prone skin, as supported by the low RCM inflammatory feature reported in our study. However, D-OCT evaluation in this study documented the reduction in vascularization, presumably through the upregulation of T-reg cells preventing inflammation, a key event in the pathogenesis of acne-prone skin [19,20]. Therefore, vascular threshold, as measured by D-OCT, may represent the objective finding of the previously assumed inflammatory milieu of acne-prone skin [7,12] and it is reasonable to assume that adjunctive probiotic supplementation in the acne-prone skin maintenance regime seems to assist in reducing increased vascularity [31].

The clinical observation of the low number of non-inflammatory acne lesions is in contrast with evidence of subclinical skin alteration, as observed by RCM and D-OCT at baseline (T0). This proves that, although not always clinically evident, acne-prone skin is a nosological entity and deserves medical attention and adequate treatment. Metaphorically, clinically visible comedones in acne-prone skin should be considered the “tip of the iceberg”. High-resolution imaging techniques prove skin alterations despite few or absent clinical manifestations.

This study is limited by its small sample size. The lack of blind methods or a health control group suggests that results should be interpreted with caution. Further, the lack of a control group with different maintenance regimes (both topical and probiotic) limited this study’s ability to infer any changes due to either the topical or probiotic therapies. Therefore, larger studies, with different comparators and a placebo-controlled cheek, are required to confirm these preliminary data.

Our preliminary study highlights subclinical alterations in acne-prone skin, despite mild clinical manifestation, and susceptibility to treatment. In patients undergoing a maintenance regime of combined topical and probiotic supplementation, a reduction in small bright follicles, inflammatory cells, vascular threshold density, and sebaceous gland hypertrophy was observed. RCM and D-OCT imaging are sensitive tools to objectively monitor subclinical skin changes.

## Figures and Tables

**Table 1:** Clinical, reflectance confocal microscopy (rcm) and dynamic optical coherence tomography (D-OCT) patient features.

Characteristic, Mean (SD)		T0, Baseline		T1, 4 Weeks		T2, 12 Weeks	
Revised Leeds Score		1.45	(0.51)	1.35	(0.49)	1.25	(0.44)
IGA grade		0.90	(0.31)	0.90	(0.31)	0.85	(0.36)
Non-inflammatory lesions		11.50	(4.25)	10.95	(3.05)	7.30 *^§^	(5.18)
	Open comedos	2.45	(2.21)	2.30	(2.25)	2.10	(1.65)
	Closed comedos	9.05	(3.87)	8.65	(2.81)	5.20 *^§^	(4.19)
RCM features							
	Small bright follicles	16.75	(6.93)	13.50 *	(3.66)	8.00 *^§^	(4.97)
	Large bright follicles	5.50	(4.26)	4.00	(4.76)	2.50 *	(4.14)
	Inflammatory features (0–3)	0.40	(0.82)	0.15	(0.37)	0.15	(0.37)
D-OCT features							
	Vascular threshold density						
	300 um	21,030.63	(10,887.40)	16,078.06	(11,465.61)	10,152.80 *	(7132.32)
	500 um	50,319.21	(26,633.76)	33,548.19	(23,747.34)	23,238.80 *	(13,232.31)
	Sebaceous gland hypertrophy (0–3)	0.74	(0.93)	0.47	(0.61)	0.42	(0.61)

RCM, reflectance confocal microscopy; D-OCT, dynamic optical coherence tomography; IGA, investigator global assessment; SD, standard deviation; * *p* value < 0.05 respect to baseline (T0) after paired Student *t*-test for continuous variables (non-inflammatory lesions, open and closed comedos, small and large bright follicles, vascular threshold density) and Wilcoxon matched-pairs signed rank test for semi-quantitative variables (revised Leeds, IGA, RCM inflammation, D-OCT sebaceous gland hypertrophy); ^§^ *p* value < 0.002 with respect to baseline (T0) after Bonferroni correction.

## Data Availability

Data is unavailable due to privacy or ethical restriction.
